# Immediate triggering of 4-1BB co-stimulation during initiation of tumor-infiltrating lymphocyte (TIL) expansion from melanoma tumors accelerates CD8+ T-cell outgrowth and tumor specificity

**DOI:** 10.1186/2051-1426-1-S1-P3

**Published:** 2013-11-07

**Authors:** Jessica Chacon, Shari Pilon-Thomas, Amod Sarnaik, Patrick Hwu, Laszlo Radvanyi

**Affiliations:** 1 Melanoma Medical Oncology, UT MD Anderson Cancer Center, Houston, TX, USA; 2 Immunology Program, UT Health Science Center Graduate School of Biomedical Sciences, Houston, TX, USA; 3 Immunology Program, H. Lee Moffitt Cancer Center and Research Institute, Tampa, FL, USA

## 

Adoptive T-cell therapy using tumor-infiltrating lymphocytes (TIL) has emerged as a powerful salvage therapy option for patients with metastatic melanoma, even those that have progressed on anti-CTLA-4 and anti-PD-1 protocols. The expansion of TIL enriched in effector-memory CD8+ T cells with demonstrated anti-tumor activity is critical in mediating durable clinical responses to TIL therapy. However, the current methods to expand TIL from melanoma tumors were not designed to facilitate the rapid and selective outgrowth of this critical CD8+ T-cell sub-population. This has required cumbersome selection techniques applied to early stage TIL cultures involving unreliable in vitro assays measuring IFN-gamma secretion in response to autologus or HLA-matched tumor cell lines. Selected early stage TIL lines are then further expanded to generate the final infusion product. Thus, a practical method to selectively expand tumor-specific CD8+ T cells from tumor fragments in situ would be a great asset. In this study, we capitalized on the ability of tumor-infiltrating CD8+ T cells to express the TNF-R family member co-stimulatory molecule 4-1BB in response to antigenic stimulation. We found that a fraction of TIL present in freshly-isolated melanoma metastases expressed 4-1BB, a sign of recent activation. Remarkably, we found that addition of an agonistic anti-4-1BB to cultures of tumor fragments from these melanoma lesions promoted early TIL outgrowth and dramatically increased the rate of CD8+ TIL expansion. These accelerated CD8+ TIL-enriched products also exhibited a much higher degree of tumor specificity, as determined in both IFN-gamma secretion and CTL assays using tumor targets. TIL initially expanded from tumor fragments with anti-4-1BB also had increased a more optimal effector-memory phenotype for adoptive transfer, including higher cytolytic granule and anti-apoptotic gene expression. Our findings suggest that 4-1BB agonists can be provided immediately during the initiation of TIL growth from tumors to develop a streamlined and practical system to accelerate the expansion of more highly tumor-specific CD8+ T cells. This approach would avoid the unreliable and cumbersome selection protocols for tumor-specificity needed for current TIL protocols and would facilitate the industrialization of TIL therapy.

